# Climate policy design, competitiveness and income distribution: A macro-micro assessment for 11 EU countries

**DOI:** 10.1016/j.eneco.2021.105538

**Published:** 2021-11

**Authors:** Toon Vandyck, Matthias Weitzel, Krzysztof Wojtowicz, Luis Rey Los Santos, Anamaria Maftei, Sara Riscado

**Affiliations:** a European Commission, Joint Research Centre, Seville, Spain; b Eurofound, Dublin, Ireland; c Banco de Portugal, Lisbon, Portugal

**Keywords:** Distributional impact, Just transition, Carbon leakage, CGE modelling, Microsimulation

## Abstract

Concerns about industry competitiveness and distributional impacts can deter ambitious climate policies. Typically, these issues are studied separately, without giving much attention to the interaction between the two. Here, we explore how carbon leakage reduction measures affect distributional outcomes across households within 11 European countries by combining an economy-wide computable general equilibrium model with a household-level microsimulation model. Quantitative simulations indicate that a free allocation of emission permits to safeguard the competitive position of energy-intensive trade-exposed industries leads to impacts that are slightly more regressive than under full auctioning. We identify three channels that contribute to this effect: higher capital and labour income; lower tax revenue for compensating low-income households; and stronger consumption price increases following from higher carbon prices needed to reach the same emissions target. While these findings suggest a competitiveness-equity trade-off, the results also show that redistributing the revenues from partial permit auctioning on an equal-per-household basis still ensures that climate policy is progressive, indicating that there is room for policy to reconcile competitiveness and equity concerns. Finally, we illustrate that indexing social benefits to consumer price changes mitigates pre-revenue-recycling impact regressivity, but is insufficient to compensate vulnerable households in the absence of other complementary measures.

## Highlights

• We quantify competitiveness-equity trade-offs in climate policy in EU countries.
• Limiting carbon leakage through free permit allocation comes at an equity penalty.
• Grandfathering is regressive by raising prices and lowering revenue for transfers.
• Impacts across households remain progressive after lump-sum revenue recycling.
• Interactions with the social benefit system affect distributional outcomes.

## Graphical abstract

**Figure:**
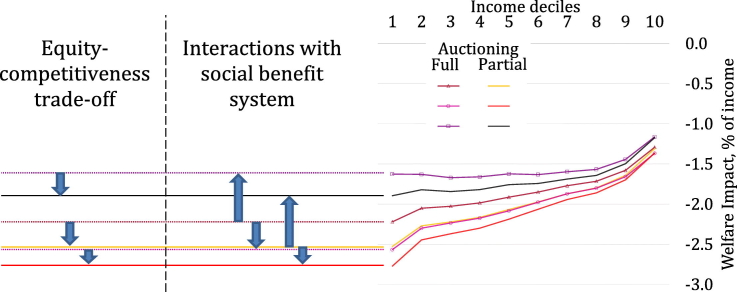

## 1 Introduction

The European Green Deal sets out an ambitious pathway to achieve climate neutrality by mid-century (European Commission, 2019a). To ensure broad-based societal support for deep decarbonisation, the corresponding policy design will need to reconcile environmental targets with concerns about competitiveness of EU industries, jobs and income inequality. Carefully designing and aligning various policy instruments (Peñasco et al., 2021) will ensure an effective policy mix for a fair transition towards a competitive low-carbon economy.

The discussion about competitiveness finds its origin in regional differentiation in climate policy. The Paris Agreement on climate change builds on a pledge-and-review system, in which countries make proposals for greenhouse gas reductions in a bottom-up fashion. This architecture allows for wide regional differences in terms of ambition levels, in contrast with the academic, stylised ideal of globally uniform carbon prices. Consequently, industries in countries with relatively strong greenhouse gas reduction targets have raised concerns about their competitive position on domestic and international markets, and modelling studies indicate potential output and export losses for emission-intensive trade-exposed sectors (Carbone and Rivers, 2017). One way to address these competitiveness concerns is to devise a climate policy that safeguards competitiveness of domestic firms. In the EU, this has taken the form of an emission permit trading scheme in which energy-intensive, trade-exposed (EITE) sectors receive a large share of permits for free, the so-called grandfathering of permits. The sectors and subsectors for which permits will be grandfathered are published on a carbon leakage list (European Commission, 2019b). Empirical work finds only limited impact of the EU Emission Trading System (ETS) on firms' competitiveness (Dechezleprêtre and Sato, 2017; Martin et al., 2016; Abrell et al., 2011) and points to a generous allocation of free permits as a likely reason (Joltreau and Sommerfeld, 2019), while theoretical work suggests that grandfathering can avert firm relocation even when phased out in the long run (Schmidt and Heitzig, 2014). At the same time, grandfathering of permits combined with cost pass-through to consumers gave rise to windfall profits, and the efficiency of permit allocation is debated (Martin et al., 2014). Other policy options, such as carbon border adjustment measures, are currently under discussion in the context of more ambitious climate policy targets in the EU Green Deal.

The importance of equity issues in climate policy relates to differences in income and expenditure patterns across households. Policies to limit greenhouse gas emissions, such as carbon prices or permit trading schemes, will raise the prices of carbon-intensive goods in order to limit their use and to encourage consumers to switch to greener alternatives. This, in turn, has led to societal concern that climate change mitigation policy would disproportionally affect low-income households that spend a higher share of their income on necessity goods, such as energy consumption for home heating. Research suggests that one way to deal with this regressivity issue is to design complementary measures to limit or offset the potential regressive effects of carbon taxes, using the extra revenue deriving from the carbon pricing (Klenert et al., 2018). For instance, whether the revenue raised by CO_2_ taxes will be recycled through labour tax cuts or by raising welfare transfers, such as unemployment benefits and pensions, may give rise to contrasting impact patterns across income groups (Vandyck and Van Regemorter, 2014; Williams III et al., 2015).

Here, we study the macro-economic and distributional impacts of climate policy in the context of the EU-wide effort to limit greenhouse gas emissions. We pay particular attention to the interaction between policy design elements that aim to address competitiveness and distributional concerns. While the decision to auction or grandfather emission permits may be primarily driven by competitiveness concerns, it may have unintended consequences for the income distribution across households. Grandfathering permits instead of auctioning may raise capital income and convince stakeholders from industry, but at the same time limits the tax revenue that is collected and available to counterbalance potential regressive impacts. Likewise, the part of tax revenue that is transferred to households to obtain a fair distribution of the impacts cannot be used to lower taxes on labour or support the industrial transformation towards carbon neutrality. Well-informed decision-making should therefore consider the interplay between these climate policy ingredients. While many papers analyse the efficiency-equity trade-offs in climate policy (see Goulder et al., 2019, for a recent example), studies of competitiveness-equity trade-offs are not commonly found in the literature. Likewise, a body of literature exists on the household distributional impacts of trade measures (see Engel et al., 2021, for a review), e.g. for Russia (Rutherford and Tarr, 2008), China (Chen and Ravallion, 2004), Nepal (Cockburn, 2006), Philippines (Bourguignon and Savard, 2008; Cororaton and Cockburn, 2007) and Mexico (Nicita, 2009). However, the literature on environmental trade measures remains largely focused on efficiency (Böhringer et al., 2017) or equity across countries (Böhringer et al., 2012) and industries (Bovenberg and Goulder, 2001). The distributional impacts of carbon leakage protection measures across households have so far received less attention. With this paper, we aim to fill some of that gap.

To explore how climate policy design can reconcile the objectives of competitiveness and equity, we compare scenarios with full and partial auctioning of emission permits in terms of macro-economic, trade and equity outcomes. Our simulations reveal a trade-off between competitiveness of energy-intensive industries and within-country equity considerations. A free allocation of permits raises the prices of carbon, domestic consumption and capital, while reducing the auction revenue that is potentially available to compensate low-income households. While all these channels act to the disadvantage of low-income households, the magnitude is limited such that progressive outcomes can still be achieved by lump-sum revenue recycling. In addition to efficiency arguments raised in earlier literature (Parry, 2003), our work therefore adds an equity-based argument for auctioning compared to free allocation of permits.

The methodological framework applied in this paper combines the economy-wide general equilibrium model JRC-GEM-E3 with the microsimulation model EUROMOD. This set-up resembles the approach of Vandyck and Van Regemorter (2014), and is further explained in the next section. The novelty of our analysis lies in the specific focus on climate policy design elements related to carbon leakage protection, in the coverage and comparison of 11 European countries, and in the assessment of the impacts of the characteristics of social benefits under different indexing schemes. Section 3 describes the scenario set-up, and Section 4 presents the results of numerical simulations. The final section provides a summary of the key insights.

## 2 Methodological framework

Economic and integrated assessment models have relied on an aggregate representation of households, but policy needs and corresponding model enhancements have led to a number of ways to bring a more refined representation of household heterogeneity into the modelling frameworks (Van Ruijven et al., 2015; Rao et al., 2017; Emmerling and Tavoni, 2021). The focus of our study calls for a modelling framework that includes multiple economic sectors and international trade on the one hand, and a rich representation of household heterogeneity on the other hand. In our set-up, we employ a computable general equilibrium (CGE) model to cover the former, and establish a top-down soft link with a microsimulation model to capture the latter dimension.

The JRC-GEM-E3 model (see Vandyck et al., 2016, for a recent application) is a CGE model designed to focus on simulations of climate and energy policy reforms. It is being used in the EU policy context to provide quantitative input into the impact assessment of a variety of policy proposals. Examples include the in-depth assessment underlying the EU long-term strategy on climate change (European Commission, 2018a) and the economic and social assessment of the proposal to increase the 2030 climate target to reduce greenhouse gas emissions by 55% below 1990 levels (European Commission, 2020). The model version developed for this exercise covers 23 sectors in the economy, including coal, crude oil, refined oil products, natural gas and electricity, disaggregated into an additional 8 electricity-generation sectors. The model is able to represent several features of the EU emissions trading system (ETS) such as grandfathered allowances and different assumptions on whether the industry can pass on opportunity costs of selling emission permits. International trade is modelled through a nested Armington (1969) specification that distinguishes between domestic and imported products on the first level, and between different exporting countries on a second level. The world is disaggregated into 42 regions, with representation of all 27 EU Member States. The model captures supply chain effects as it is based on input-output tables from the GTAP 9.2 data set (Aguiar et al., 2016; see van Tongeren et al., 2017 for a historic perspective) with disaggregated power sectors (Peters, 2016). Energy use and the corresponding CO_2_ emissions are modelled as inputs into the production process on the firm side, and as fuel consumption linked to the use of two types of durables – housing and vehicles – on the household side. For the simulations in this paper, we assume that both labour and the stock of capital are mobile across sectors but immobile across countries.

EUROMOD (see Barrios et al., 2019, for a recent application) is a static microsimulation model for the EU used to compute tax liabilities and household disposable income. It captures the richness of household heterogeneity in terms of income and other socio-economic characteristics, allowing for interactions between the components of the tax and benefit system. The EUROMOD-ITT version (De Agostini et al., 2017) that we use here adds to the general version of this microsimulation model by considering also household expenditure patterns obtained by matching expenditure (from the Household Budget Survey) with income (from EU-SILC) data. We use the model here under the assumption of constant quantities, which implies that the distributional analysis does not consider that the behavioural response to price changes may differ across households with different socio-economic characteristics. For instance, our modelling framework does capture liquidity constraints for low-income households to invest in energy-efficient infrastructure when energy prices rise. The results presented in this paper cover 11 of the 27 current EU Member States that were covered by EUROMOD-ITT at the time of performing this analysis: Austria (AUT), Belgium (BEL), Czech Republic (CZE), Estonia (EST), Finland (FIN), France (FRA), Germany (DEU), Greece (GRC), Italy (ITA), Romania (ROU) and Spain (ESP). Jointly, they represent over 70% of the EU population and CO_2_ emissions. Although this sample covers countries with a wide variety in incomes and energy systems, results cannot be automatically generalised to other EU countries that are not covered here.

We soft-link both models in a top-down fashion, by first running the CGE model, and then providing results as inputs to the microsimulation analysis. This is a unidirectional link, and no information is passed on from the micro level to the CGE model. In particular, 17 variables are passed on from the CGE to the microsimulation model for each country and scenario: consumption prices changes for 14 categories, factor price changes for two income sources, and additional tax revenue that can be redistributed to households. On the expenditure side, we derive climate policy-induced price changes, relative to baseline levels, for 14 consumption good categories (see Supplementary Table 1). These price changes include both direct effects of carbon prices on household energy use and indirect price changes through intermediate inputs along the supply chain. One of the advantages of using a general equilibrium model in this exercise is that we do not need to assume full pass-through of carbon prices, as we model the supply side of the economy explicitly, i.e. the climate policy-induced prices result from the economic agents' optimizing behaviour including mitigation. One of the challenges in linking the two models is that the input-output tables and the expenditure data use a different statistical classification. We overcome this issue by including a bridging or consumption matrix in the CGE model that translates the classification of producing sectors or economic activities into the Classification of Individual Consumption by Purpose (COICOP). This is particularly relevant for the work we present here, as this feature facilitates the link with the microdata in the Household Budget Survey, which contains expenditure patterns in the COICOP classification for products. Recent bridging matrices for all EU countries have been made available publicly for all EU countries by Cai and Vandyck (2020). Further details on the mapping of consumption categories is presented in Supplementary Table 1. On the household income side, the relative changes to the baseline for both labour and capital remunerations serve as inputs for the microsimulation. More specifically, the percentage changes in wage rates and returns to capital are applied to the corresponding income components from the EU-SILC data when implementing the different policy alternatives. For the purpose of this paper, the CGE model assumes flexible wages, such that unemployment rates remain at the level of the baseline and labour market impacts are fully captured and passed on to the microsimulation through wage adjustment. For a discussion of alternative approaches, we refer to Hérault (2010) and Bargain et al. (2012). Finally, the absolute tax revenue that can be handed out as a lump-sum compensation to all households, after general equilibrium interactions and assuming budget neutrality, is passed on to the microsimulation model. This tax revenue is adjusted by the income growth between 2015 and 2030 to account for the fact that we pass on 2030 macro results to a static microsimulation with 2015 micro data. In the default scenarios we assume that household income from social benefits (e.g. unemployment, sickness and pension benefits) is fixed in nominal terms, but we explore alternative assumptions in Section 4.2.

The soft-link approach described above has clear merits, as it allows combining two specialised and complementary models to provide a more comprehensive assessment. In contrast to a hard-linked framework, however, our top-down soft-link approach leaves unaddressed some inconsistencies both in the initial datasets and in the behavioural responses. For instance, levels and shares of income and expenditure categories in the two models will not match exactly. In addition, the CGE model puts quite some effort in developing a baseline up to the year 2030, while the microsimulation provides a static view of the base year. As a result, the welfare impacts as calculated by the microsimulation, when aggregated over all households, are unlikely to match the CGE outcomes. This can give rise to an ambiguity in the interpretation of results. Therefore, we take the aggregate CGE welfare impact as given in this exercise, and use the microsimulation model to translate this into impacts for different household groups. To harmonise aggregate welfare impacts (relative to the baseline) for each country, we add a final step to the analysis. In this step, we use a homogeneous adjustment factor across all income groups to obtain the same aggregate welfare impact (relative to income) per country as for the representative household in the CGE analysis. In particular, on the micro level we monetise welfare impacts *Δy*^*h*^ for a household *h* by applying relative consumption price changes *Δp*_*i*_/*p*_*i*_ for goods *i*, relative factor price changes *Δr*/*r* and *Δw*/*w* (for capital and labour endowments, *K*^*h*^ and *L*^*h*^, respectively) and transfer income changes *ΔT*^*h*^ according to the following formula:

(1) $$\Delta y^{h}=rK^{h}\frac{\mathrm{\Delta r}}{r}+{\mathrm{wL}}^{h}\frac{\mathrm{\Delta w}}{w}+\Delta T^{h}-\sum_{i=1}^{14}p_{i}q_{i}^{h}\frac{{\mathrm{\Delta p}}_{i}}{p_{i}}$$

This equation expresses welfare impacts assuming ‘fixed quantities’, representing the immediate effect when a households' endowments *K*^*h*^ and *L*^*h*^ and consumption choices *q*_*i*_^*h*^ remain unchanged. We then harmonise aggregated welfare impacts with the outcomes of the macro model, choosing the Compensating Variation for the representative household (*Δy*^*RH*^) as the welfare metric, by using an adjustment factor *d* that is defined by the following equation:

(2) $$\underset{\mathrm{JRC}-\mathrm{GEM}-E3}{\underbrace{1+\frac{\Delta y^{\mathrm{RH}}}{y_{0}^{\mathrm{RH}}}}}=d\underset{\mathrm{EUROMOD}-\mathrm{ITT}}{\underbrace{\left(1+\frac{\sum_{h}\Delta y^{h}}{\sum_{h}y_{0}^{h}}\right)}}$$

In this equation, *y*_0_ denotes pre-reform disposable income. We then calculate macro-consistent micro impacts *Δy*^′*h*^ by applying the adjustment factor *d* to relative welfare impacts at the household level (*Δy*^*h*^/*y*_0_^*h*^) such that the following holds:

(3) $$1+\frac{\Delta y^{\prime h}}{y_{0}^{h}}=d\left(1+\frac{\Delta y^{h}}{y_{0}^{h}}\right)$$

This adjustment procedure brings the advantage that we can now readily interpret the microsimulation analysis as a breakdown of the CGE model welfare impacts into household income groups. The downside is that this rescaling approach is agnostic about the source of the underlying data inconsistency, and could introduce a bias to the results if differences in aggregate impact arise from a particular factor that is systematically skewed towards one end of the income distribution. For instance, the rescaling could bias results if energy efficiency investments in the period 2015–2030 are concentrated in low-income households, or when aggregate impact differences would be largely attributed to changes in the return to capital, which is typically underreported in micro-level survey data.

**Fig. 1:**
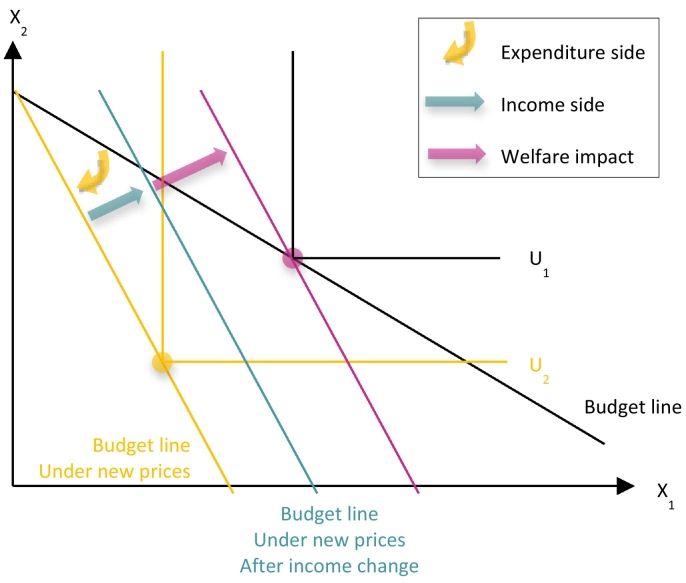
Stylised representation of the different components of the link between macro and micro model and the Compensating Variation welfare concept used to harmonise aggregate impacts between the two models.

The different components in the micro-level analysis can then be summarised using the stylised representation in Fig. 1. The assumption of constant quantities in EUROMOD-ITT is here interpreted as derived from Leontief preferences (U_1_), since households will be consuming exactly the same basket of goods before and after the policy shock. The policy reform leads to an increase in the price of a given good *X*_*1*_, for instance energy. This price change alters the slope of the budget line, as indicated by the yellow arrow. Income changes, for instance due to a transfer of tax revenue, shifts the budget line out (green arrow). Finally, the purple arrow indicates the monetary transfer that is necessary to bring the household to the pre-reform utility level, given the new prices. This concept matches the definition of Compensating Variation as a money metric of the welfare change. In the remainder of the paper, we choose to represent detrimental welfare impacts with negative numbers, as this will be more intuitive for the majority of readers.

## 3 Scenarios

For this paper, we develop one baseline and two climate policy scenarios, all of which are rooted in the Energy Modelling Forum (EMF) 36 on “Carbon Pricing After Paris” (Böhringer et al., 2021). In the baseline, global energy-related CO_2_ emissions and GDP are calibrated to the World Energy Outlook of IEA (2018) using the PIRAMID framework (Wojtowicz et al., 2019). This approach first projects input-output tables based on the initial GTAP input-output tables, and calibrates the CGE model parameters to this exogenous time series of input-output tables in a second step. The projection targets GDP and emission assumptions from IEA (2018) in line with the scenario protocol for EMF 36. In addition, we take into account energy modelling output to capture energy system trends, such as changes in the fuel mix, as described in Wojtowicz et al. (2019). Exogenous energy data was scaled appropriately to reproduce IEA emission projections. The first scenario considers ***Full Auctioning*** of permits, with uniform permit prices across countries and sectors in the EU rising to approximately 100 USD/tCO_2_ in 2030. This price is around 3.5 times higher than the global average carbon price (see below), which could justify free allowances with a competitiveness motive. The second, ***Partial Auctioning*** scenario considers grandfathering of all permits in the energy-intensive trade-exposed (EITE) sectors covered by the EU Emission Trading System (except for power generation, where we assume that all permits are auctioned in both scenarios). Greenhouse gas emissions are kept at the levels of the *Full Auctioning* scenario at Europe-wide level. As partial auctioning is less effective than full auctioning in reducing emissions in EITE sectors, the implied carbon price is 17% higher than in the *Full Auctioning* scenario.

In these climate policy scenarios, CO_2_ emission constraints are implemented in the JRC-GEM-E3 model such that countries meet the Nationally Determined Contributions (NDCs) submitted to the UNFCCC (see Böhringer et al., 2021, for a more detailed description and derivation of targets). For an NDC scenario, carbon prices are relatively high because we run the model in stand-alone mode and use the carbon price as the only policy instrument. In comparison to the typical set-up for policy assessment, where we link the model with an energy system model, this results in a fairly stylised representation of energy technology processes. A more elaborate approach, where the model is linked with detailed bottom-up energy models, typically results in lower carbon prices, as penetration of technologies with initially low shares (e.g. electric vehicles) tends to be underestimated in the CGE model compared to the bottom-up energy system models. In addition, real-world climate policies include elements other than carbon pricing, such as renewable portfolio or energy efficiency standards, which may lower carbon prices (and corresponding tax revenue) required to achieve a specified emission reduction goal. We furthermore abstract from other elements in EU climate policy, particularly the split between ETS and non-ETS sectors, and the corresponding Effort Sharing Regulation for the latter. Our focus lies on within-country distributional impacts (and the interaction with carbon leakage protection measures) in a situation where carbon price signals apply throughout the economy. While we do not capture all the details of current policy implementation, our scenarios are policy-relevant as an extension of the ETS to cover buildings and transport is currently under discussion, and various Member States have already implemented carbon pricing domestically. For an in-depth analysis of between- and within-country distributional impacts, we refer to the work of Landis et al. (2021). Finally, the model version used in this paper only represents combustion emissions from fossil fuels, abstracting from industrial process emissions and non-CO_2_ greenhouse gases, which can constitute low-cost abatement options (Weitzel et al., 2019). The distributional impacts thus have to be seen in the context of this stylized set-up. Taxes on non-CO_2_ greenhouse gas emissions may particularly affect food prices and could yield regressive impacts (Kehlbacher et al., 2016).

## 4 Results and discussion

To understand the mechanisms behind the key findings of the paper, we follow the sequence of the methodological approach in the presentation of results. After describing the economy-wide and competitiveness effects of both climate policy options, and the resulting price changes that feed into the micro-level analysis (section 4.1), we dive into the impact heterogeneity across household income groups (section 4.2).

### 4.1 Multi-sector macro-economic outcomes

GDP impacts of climate policy in the European countries considered range between 0.1% and − 0.5% in 2030 relative to baseline levels (Table 1). To put this into perspective, note that the latter number would be equivalent to a change in annual GDP growth rate from 2% in the baseline to 1.95% in the scenario for the period 2020–2030. Welfare impacts, presented as compensating variation as a share of income, have the same order of magnitude. For both GDP and welfare, the differences between the two scenarios are generally small. While GDP impacts can be slightly higher or lower under Partial Auctioning compared to Full Auctioning, welfare losses are consistently larger than in the Full Auctioning scenario. The Partial Auctioning scenario improves international competitiveness for sectors that receive free allowances and the balance of trade can adjust accordingly. To accommodate higher output in energy-intensive trade-exposed (EITE) sectors while maintaining the aggregate emission constraint for Europe at the level of the Full Auctioning scenario, other sectors have to abate more and the EU-wide carbon price rises compared to the Full Auctioning scenario. Correspondingly, welfare losses are higher under the Partial Auctioning scenario. The effect on GDP in an individual country can go in either direction, depending on the share of EITE sectors in the country's GDP.

**Table 1:** Macro and sector-specific impacts of climate policy. The first number in a cell indicates the Full Auctioning scenario in 2030 and the second one represent the Partial Auctioning scenario (in percentage changes from the baseline). Source: JRC-GEM-E3 model.

Full/Partial auction	AUT	BEL	CZE	DEU	ESP	EST	FIN	FRA	ITA	GRC	ROU
GDP	−0.06/ −0.05	−0.14/ −0.14	−0.16/ −0.14	−0.33/ −0.35	0.01/ 0.01	−0.49/ −0.51	−0.08 /−0.07	−0.07/ −0.06	−0.27/ −0.28	−0.15/ −0.06	−0.02/ 0.07

Welfare	−0.16/ −0.16	−0.06/ −0.08	−0.48/ −0.52	−0.35 /−0.39	−0.15/ −0.16	−0.65/ −0.69	−0.21/ −0.26	−0.17/ −0.19	−0.35/ −0.38	−0.39/ −0.29	−0.35/ −0.36

Output											
Fossil fuels	−6.5/−3.9	−4.6/−3.3	−16/−14	−9.0/−6.8	−10/−4.3	−30/−34	−8.5/−3.2	−6.8/−1.3	−8.4/−5.8	−23/−13	−16/−8.6
EITE	−0.7/0.7	0.0/0.2	−3.5/−1.2	−2.0/−1.4	0.9/1.1	−0.6/−0.8	−1.9/−0.2	0.6/1.3	−1.4/−1.5	−0.9/0.2	−5.2/−0.6
Manufacturing	−0.1/−0.2	−0.1/−0.2	−0.2/−0.2	−0.2/−0.3	0.0/−0.1	−4.4/−4.6	0.1/−0.1	0.0/−0.2	0.0/0.0	−0.6/−0.6	0.3/0.2
Agriculture	0.6/0.2	−0.1/−0.2	2.3/2.0	2.1/2.1	−0.1/−0.4	2.2/2.6	0.3/0.2	−0.4/−0.6	0.3/0.2	0.6/0.4	0.8/0.3
Transport	−0.4/−0.1	−1.8/−1.4	−1.8/−0.9	−1.1/−0.3	−4.0/−1.5	−1.7/−1.5	−2.2/−0.5	−2.5/−1.1	−1.7/−1.0	−23/−21	−1.7/−1.0
Services	−0.1/−0.1	−0.1/−0.1	0.1/0.1	−0.1/−0.2	−0.1/−0.1	0.2/0.2	0.0/−0.1	−0.1/−0.1	−0.1/−0.1	0.0/0.0	−0.3/−0.2

Export											
Fossil fuels	−9.5/−5.3	−4.8/−2.9	−20/−19	−9.7/−5.8	−9.0/0.1	−28/−33	−6.3/0.1	−8.4/−0.6	−9.7/−5.9	−18/−0.6	−23/−2.9
EITE	−1.0/0.7	0.0/0.2	−4.0/−1.5	−2.3/−1.6	1.6/1.8	−0.6/−0.8	−2.3/−0.3	0.9/1.8	−2.0/−2.1	−1.3/0.3	−9.0/−1.5
Manufacturing	0.0/−0.4	−0.3/−0.4	−0.1/−0.4	−0.1/−0.4	−0.3/−0.7	−1.8/−1.7	0.7/−0.2	0.0/−0.5	−0.3/−0.4	−3.0/−3.6	1.8/0.4
Agriculture	1.0/0.2	−0.5/−0.6	1.9/1.0	2.8/2.7	−0.6/−1.4	−0.7/−0.5	0.4/−1.2	−1.2/−1.9	0.6/0.4	2.2/1.3	1.9/0.3
Transport	−0.3/0.0	−2.9/−2.3	−2.8/−1.2	−1.9/0.0	−11/−3.6	−2.3/−1.9	−4.2/0.6	−5.3/−1.6	−5.9/−1.7	−33/−31	−3.9/−1.3
Services	−0.4/−1.2	−0.8/−1.0	2.3/1.3	0.3/−0.1	0.1/−0.3	1.9/2.1	0.3/−0.5	−0.6/−1.0	0.8/0.7	0.4/−0.1	0.0/−1.0

Import											
Fossil fuels	−6.8/−5.4	−5.8/−5.3	−9.2/−7.7	−6.6/−6.0	−9.3/−6.1	−4.4/−3.5	−8.2/−5.4	−5.7/−3.9	−8.0/−7.3	−15/−12	−11/−7.6
EITE	−0.6/−0.2	−0.2/−0.1	−1.0/−0.5	−0.5/−0.3	−0.6/−0.5	−0.8/−0.7	−0.5/0.0	−0.3/−0.3	−0.6/−0.4	−0.3/−0.4	−0.6/−0.6
Manufacturing	−0.2/−0.2	−0.2/−0.2	−0.7/−0.6	−0.4/−0.4	−0.2/−0.1	−1.2/−1.2	−0.3/−0.1	−0.2/−0.2	−0.5/−0.5	−0.5/−0.2	−1.0/−0.5
Agriculture	0.2/0.2	−0.2/−0.2	0.3/0.4	−0.6/−0.6	−0.1/0.1	0.2/0.4	0.5/1.2	−0.1/−0.1	0.4/0.4	−1.4/−1.2	−1.4/−1.2
Transport	−0.4/0.1	−1.4/−0.6	−1.8/−0.5	−1.2/−0.5	−4.6/−0.1	−1.4/−0.9	−1.6/−0.4	−2.1/−0.4	−1.8/−0.7	−14/−14	−1.6/−0.7
Services	0.1/0.4	0.3/0.3	−1.1/−0.7	−0.4/−0.3	−0.2/0.0	−0.9/−1.0	−0.2/0.1	0.2/0.3	−0.5/−0.6	−0.1/0.2	−0.2/0.3

The results on sectoral output indicate, unsurprisingly, that the major impacts occur in the fossil fuel-producing sectors, with output losses across countries ranging between 1% and 35%. The sectors grouped under ‘energy-intensive trade-exposed (EITE) sectors’ experience aggregated output changes between −5% in Romania and + 1% in France. Importantly, these sectors tend to have higher output levels when permits are grandfathered, particularly in countries where production is more emission-intensive, such as Czech Republic and Romania. On average, Partial Auctioning limits output losses in the EITE sectors by more than 1 percentage point compared to Full Auctioning, indicating that the grandfathering of permits is beneficial for the EITE sectors. Changes are more pronounced on export markets, where free allowances increase international competitiveness. The most striking example can be observed for Romania, where the impact of climate policy on exports of the EITE sectors of −9% relative to baseline under Full Auctioning is limited to just −1.5% when these sectors receive the emission permits for free. Stronger foreign demand may raise prices on the domestic market, such that imports tend to go up moderately compared to the case of Full Auctioning.

Carbon prices affect sectors and goods according to emission intensity of their production. Table 2 reports the climate policy-induced price changes for the consumption categories that remain after matching JRC-GEM-E3 product disaggregation with the one of the EUROMOD-ITT model. As expected, the prices of energy-intensive consumption categories related to residential fuel use and transport (in bold) experience the largest price increases. The category of Home fuels covers residential energy use, including expenses for heating and electricity. The share of fossil fuels in residential heating and electricity generation can be an important driver of price impact variation across countries. The category of Private transport is not restricted to fuel use, but also covers maintenance and insurance related to the operation of a vehicle. Typically, higher income countries have a lower share of fuels in the consumption categories, hence relative price increases from the same carbon price are lower. Furthermore, higher income countries often have higher excise taxes on fossil fuels, which dampens the relative price increase in these countries. The category of Public transport is generally less carbon-intensive than Private transport, as it includes the consumption of related services. For Greece, the transport sectors experience a stronger price increase than in other countries, since these sectors are comparably very fuel intensive and contain a relatively high share of waterborne transport, which has a high carbon intensity per monetary value compared to other transport modes.

**Table 2:** The impact of climate policy on prices. The first number in a cell indicates the Full Auctioning scenario in 2030 and the second one the Partial Auctioning scenario (in percentage changes from the baseline). Bold numbers highlight energy-related goods. Source: JRC-GEM-E3 model.

Full/Partial auction	AUT	BEL	CZE	DEU	ESP	EST	FIN	FRA	ITA	GRC	ROU
Food	0.6/0.8	0.8/0.9	0.3/0.6	0.6/0.7	0.5/0.7	0.9/1.0	0.7/0.9	0.7/0.8	0.5/0.5	0.5/0.7	0.3/0.6
Clothing	0.6/0.8	0.8/0.9	0.3/0.5	0.6/0.7	0.5/0.6	0.9/1.0	0.7/0.9	0.6/0.8	0.4/0.5	0.6/0.7	0.4/0.7
**Home fuels**	**7.4/8.6**	**11/13**	**16/18**	**13/15**	**4.9/5.5**	**18/19**	**6.3/7.1**	**4.8/5.5**	**12/14**	**8.6/9.2**	**15/17**
Rents	0.4/0.6	0.5/0.6	−0.3/0.0	0.2/0.4	0.3/0.4	−0.2/−0.2	0.3/0.5	0.4/0.6	0.1/0.2	0.3/0.5	0.4/0.7
Household goods	0.7/0.7	0.8/0.8	0.6/0.7	0.6/0.6	0.5/0.6	0.7/0.7	0.6/0.7	0.7/0.7	0.5/0.5	0.5/0.6	0.6/0.7
Health	0.6/0.7	0.7/0.7	0.7/0.7	0.4/0.5	0.4/0.5	0.9/0.8	0.4/0.5	0.5/0.6	0.3/0.3	0.3/0.4	0.9/0.8
**Private transport**	**4.4/4.9**	**3.9/4.3**	**12/13**	**5.3/5.9**	**8.1/9.0**	**13/15**	**7.7/8.6**	**5.2/5.8**	**4.7/5.3**	**21/23**	**6.5/7.1**
**Public Transport**	**3.1/2.1**	**3.2/2.4**	**3.1/3.1**	**3.9/2.2**	**6.7/4.2**	**3.6/3.5**	**3.9/3.2**	**4.4/3.0**	**4.9/3.8**	**12/7.6**	**3.2/3.4**
Communication	0.3/0.6	0.5/0.6	−0.3/0.0	0.2/0.4	0.2/0.4	−0.1/−0.1	0.3/0.5	0.4/0.6	0.1/0.2	0.2/0.4	0.3/0.6
Recreation	0.6/0.8	0.8/0.8	0.0/0.3	0.3/0.5	0.5/0.7	0.3/0.4	0.5/0.7	0.7/0.8	0.4/0.5	1.6/1.8	0.7/1.0
Education	0.4/0.6	0.4/0.5	0.0/0.3	0.1/0.3	0.2/0.3	0.1/0.0	0.2/0.4	0.3/0.5	0.0/0.1	−0.1/0.1	−0.2/0.2
Restaurants	0.6/0.8	0.8/0.8	0.0/0.3	0.3/0.5	0.5/0.7	0.3/0.4	0.5/0.7	0.7/0.8	0.4/0.5	1.6/1.8	0.7/1.0
Other	0.5/0.6	0.6/0.7	0.0/0.2	0.4/0.5	0.3/0.4	0.2/0.2	0.5/0.6	0.5/0.6	0.2/0.3	0.3/0.5	0.4/0.6
Durable goods	0.7/0.8	0.9/0.9	0.7/0.7	0.7/0.7	0.6/0.7	0.7/0.7	0.7/0.8	0.8/0.8	0.6/0.6	0.5/0.6	0.7/0.8
											
Labour income	0.0/0.3	0.1/0.2	−1.0/−0.6	−0.4/−0.3	−0.1/0.1	−0.7/−0.8	−0.3/0.0	0.1/0.3	−0.5/−0.5	−0.5/−0.3	−0.6/−0.2
Capital income	0.0/0.3	0.1/0.2	−1.0/−0.7	−0.3/−0.2	−0.1/0.1	−1.4/−1.4	−0.3/−0.1	0.1/0.3	−0.5/−0.4	−0.5/−0.2	−0.4/0.0
CPI	0.7/0.8	0.8/0.8	0.6/0.8	0.8/0.8	0.6/0.7	1.6/1.6	0.7/0.8	0.7/0.8	0.7/0.7	1.2/1.2	0.7/0.9
Auction revenue (billion $)	3.2/2.9	5.9/5.8	3.6/3.6	36/33	15/14	0.6/0.7	2.9/2.5	22/20	23/24	5.7/5.8	3.8/3.7

The carbon policy affects not only consumption prices, but also households' incomes. On the one hand, as firms move away from energy inputs into their production, capital and labour become more attractive production factors. On the other hand, some sectors contract as their output declines, resulting in lower factor demand. This translates to changes of capital and labour prices shown in the bottom rows of Table 2. Assuming fixed households' factor endowments and flexible factor prices (no unused capital and unemployment rates fixed at baseline levels), this leads to a change of household income in nominal terms. When compared against consumption price changes, it becomes clear that factor income decreases in real terms in all countries. The relative change in capital and labour prices can also contribute to how different income groups are affected by the climate policy as higher income households derive a larger share of their income from capital income (see section 4.2 below). A common observation in the literature is that return on capital declines more than labour prices, although the differences are not necessarily very strong (e.g., Rausch et al., 2011; Dissou and Siddiqui, 2014; Williams III et al., 2015; Goulder et al., 2019). Goulder (1995) argues that carbon taxes could raise the cost of producing capital goods, thus reducing the rate of return. As a recursive-dynamic model, JRC-GEM-E3 does not optimize investment and consumption pathways intertemporally, however, investment demand is influenced by the return to capital and the cost of capital goods. This typically leads to increased investment in climate policy scenarios. In addition to this supply-side argument for the production of capital goods, climate policy will also affect the demand for capital goods. If capital-intensive sectors (such as the EITE sectors) have a stronger decline in output than labour-intensive sectors (such as services), this will lead to a shift from capital to labour and reduce the capital price relative to the labour price. However, there could also be shifts *within* sectors to abate fossil energy use by using more capital-intensive equipment, potentially offsetting the shift of the economy to less capital-intensive production sectors. In comparison to the papers listed above, JRC-GEM-E3 has a more disaggregated technology representation. In response to a carbon price in the electricity sector, the model therefore tends to rather substitute to clean electricity than switching away from electricity use, increasing the capital intensity rather than decreasing it. A cleaner electricity mix then also allows increased electrification of other sectors, further increasing the demand for this capital-intensive sector. This also corresponds to the literature quantifying the additional investment needs to decarbonize the energy system (McCollum et al., 2018). In addition to capital and labour income, the revenues of the auctioning of emission permits affect household incomes as we assume these revenues are recycled in the form of lump-sum transfers to households. Note that we abstract here from effort-sharing agreements and from transfers of tax revenues between European countries. For simplicity, we assume that revenues from taxing emissions in a country are recycled in full to households in that country. While some of the permits are grandfathered in the Partial Auctioning scenario, the carbon price is higher to maintain the same level of emissions, such that the change in auction revenues across scenarios is relatively small.

### 4.2 Household heterogeneity: distributional impacts

**Fig. 2:**
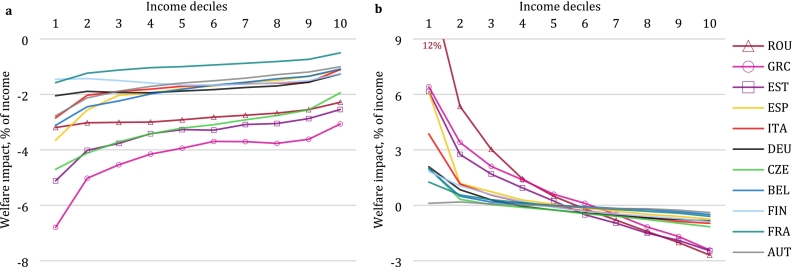
Welfare impact by income decile (Full Auctioning scenario). a, Impact due to changes in consumption prices and in labour and capital income before lump sum transfer. b, Full impact including lump sum transfer of tax revenue and rescaling to be consistent with CGE model aggregate welfare impacts. Welfare impacts are shown as Compensating Variation as a percent of disposable income. Source: EUROMOD-ITT extension.

The consumption and factor price changes presented in the previous section form the basis for the distributional analysis. Fig. 2 illustrates the welfare impacts for 10 income deciles in the 11 European countries covered in our study, assuming Full Auctioning of emission permits. The figure decomposes the total welfare effect into impacts before (panel a) and after (panel b) recycling of additional revenue from permit auctioning in the form of lump-sum transfers to households. The impacts before revenue recycling show a regressive pattern, with higher effects relative to income for the lower income deciles. The results in Fig. 2a stem from both consumption and factor (capital and labour) price changes. However, redistributing the permit auction revenue on an equal-per-household basis reverts the pattern and turns the full effect of the reform into a progressive one (panel b), as poorer households gain welfare relative to the baseline while the transfer is too small relative to income to compensate for incurred welfare losses for the high-income deciles.

The impacts before revenue recycling are relatively large for Greece and Estonia, where we observe the largest increase in consumer prices. We observe effects of comparable magnitude in the Czech Republic, which has the largest drop in factor prices. However, considering the full effect including revenue recycling, these countries are not necessarily worse off than other countries. This indicates that in these countries, carbon pricing might translate to higher expenditures, but also higher revenues from carbon pricing that can offset negative effects. Taking into account the full effect shows the highest progressivity for Romania, Greece and Estonia – countries with lower per capita income. Their low-income deciles are thus among the poorest households in Europe (Temursho et al., 2020). In our analysis, these households are found to benefit most, despite not assuming international transfers between countries as in the allocation of auctioning revenues under the current EU ETS implementation. Assuming lump-sum revenue recycling, adding transfers from high- to low-income EU Member States to the analysis would strengthen the progressive impact pattern in the transfer-receiving countries.

**Fig. 3:**
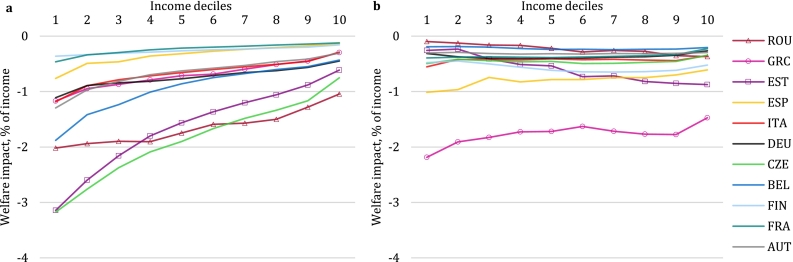
Decomposition of expenditure side impacts related to energy goods (Full Auctioning scenario, before revenue recycling). a, Residential energy use. b, Transport. Welfare impacts are shown as Compensating Variation as a percent of disposable income. Source: EUROMOD-ITT extension.

To gain insight into what is driving the distributional patterns, we further investigate individual components of the expenditure-side effect. We isolate the impact of price changes for residential and transport energy by passing only price changes for these individual consumption categories to EUROMOD-ITT. Fig. 3 illustrates that the regressive expenditure-side impacts due to residential energy use, for which expenditures take up a higher share of disposable income for the low-income households, are typically stronger than the effects driven by fuel-related transport expenditures. The strong welfare impact in the Czech Republic can be explained as average expenditures for household fuels are high and the renewable share of heating and cooling fuels is low (Supplementary Fig. 1). Effects in Romania and Estonia are driven by a price increase that exceeds the European average. However, the relative importance of distinct energy uses differs across countries: in Spain and Greece, Southern European countries with lower residential heating needs, the effects of transport expenditures appear more pronounced than those of heating energy. While residential energy price changes lead to regressive effects in all countries, transport impacts show a flat or even progressive pattern in some countries, such as Estonia. A notable exception is Greece, which has both larger absolute welfare impacts from price changes in transport related to the above average price increases compared to other countries (Table 2), as well as a mild regressive effect.

**Fig. 4:**
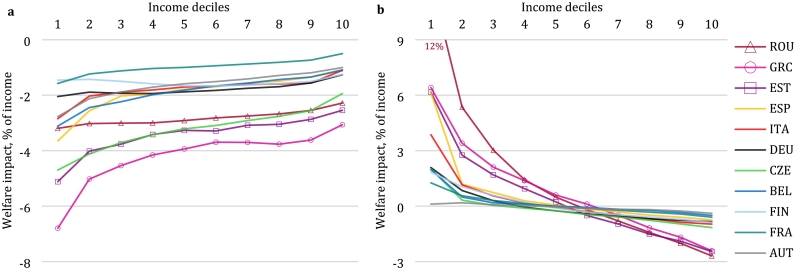
Impact of climate policy design. a, Impact due to changes in consumption prices and in labour and capital income before lump sum transfer. b, Full impact including lump sum transfer of tax revenue and rescaling to be consistent with CGE model outcomes. Negative values indicate that the Partial Auctioning scenario with grandfathering has lower welfare levels than under Full Auctioning for that income decile. Source: EUROMOD-ITT extension.

Results have so far focused only on the Full Auctioning scenario. As illustrated in Section 4.1, grandfathering instead of auctioning permits in the energy-intensive trade-exposed sectors improves their competitive position on international markets and correspondingly raises exports. The effect on distributional outcomes is unclear a priori. Fig. 4 reveals how welfare impacts across income deciles change (percentage point difference from Full Auctioning) when permits are grandfathered instead of auctioned in the EITE sectors. Output levels and hence emissions in EITE sectors are higher under grandfathering, hence a stronger carbon price signal is needed to induce further abatement, leading to a carbon price about 17% higher than in the case of Full Auctioning. As a result, the observed price increases are stronger in the Partial Auctioning scenario (Table 2), causing more regressive effects before revenue recycling (Fig. 4a). In terms of emission permit auctioning revenue, two counteracting factors are at play: on the one side, only auctioning part of the permits shrinks the tax base, but on the other hand, tax rates to meet the emission constraints are higher. For most countries, the tax base effect prevails, slightly reducing carbon tax revenue. This implies that fewer resources are available for lump-sum revenue recycling, limiting the positive impact from revenue recycling on low-income households under Partial Auctioning compared to Full Auctioning (Fig. 4b). While the size of the difference in welfare impact is about one order of magnitude smaller than the welfare losses before revenue recycling (Fig. 2a), it can lead to a sign switch in instances where final impacts under Full Auctioning were small, such as the first income decile in Austria (see Fig. 2b). However, the tax rate effect comes out more strongly in Estonia, Italy and Greece. In these countries, the output share of energy-intensive trade-exposed sectors is lower than the EU average (of around 10% in 2030), which suggests that the tax base effect plays less of a role here. The tax rate effect is equal for all countries as they share a common price under the EU ETS system. These results highlight some of the complexities related to fiscal federalism when emission-trading systems cover a heterogeneous set of countries. Due to the higher tax revenue under Partial Auctioning, Estonia shows more progressive impacts than under Full Auctioning, while for Greece and Italy the extra revenues under Partial Auctioning roughly offset the additional regressivity from consumption and factor price changes compared to the Full Auctioning case.

Finally, we explore the sensitivity of the results with respect to the indexation schemes for social benefits, such as unemployment benefits and pensions. In these sensitivity tests, we disregard the transfer of permit auctioning revenue, as we are interested here in how more general social payment schemes interact with climate policy and influence the distributional outcomes. We furthermore assume that the social benefit indexation is financed from the general government budget and ignore general equilibrium feedbacks in this sensitivity analysis. Social benefit systems differ across countries in the EU, with social benefits typically tied in some form to the consumer price index (CPI), general evolution of wages, or a combination of both (European Commission, 2018b). Results shown in previous figures accounted for the fact that some households may become entitled to certain means-tested benefits when household income drops. These changes in eligibility for social benefits are often overlooked in academic studies. However, we had assumed that no indexation takes place, such that the level of any particular type of social benefit remains at the level of the baseline. Here, we relax that assumption by studying CPI- or wage-based indexation schemes for all social transfers combined, while keeping eligibility unchanged. As highlighted by Goulder et al. (2019) in the context of the US, assumptions on social transfer indexation can play a significant role in determining the distributional pattern of impacts across household income groups.

**Fig. 5:**
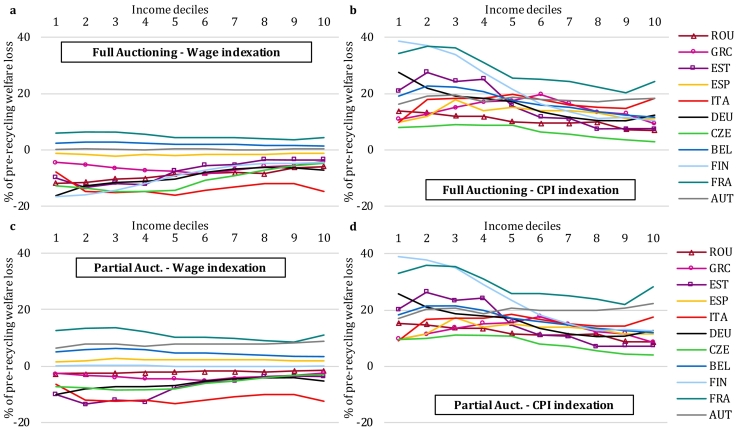
Welfare loss offsetting under different indexation schemes for social transfers and benefits. a, Full Auctioning, wage-based indexation of social benefits. b, Full Auctioning, CPI-based indexation of social benefits. c, Partial Auctioning, wage-based indexation of social benefits. d, Partial Auctioning, CPI-based indexation of social benefits. Source: EUROMOD-ITT extension.

Fig. 5 illustrates the extent to which the social benefit system offsets the (pre-revenue-recycling) welfare losses incurred by households in our policy scenarios. In this figure, a positive value of, say, +10%, would indicate that 10% of the decile's welfare loss resulting from changes in consumption and factor prices is mitigated by changes in the level of social benefit transfers. A number of insights emerge.

First, results indicate that the share of the (pre-revenue-recycling) welfare loss that is potentially offset by social benefit indexation ranges between −20% and + 40% across deciles, scenarios and countries considered (−16% in the fifth income decile in Italy under Full Auctioning and Wage Indexation; +38% in the first income decile in Finland under Partial Auctioning and CPI indexation). As such, generic rule-based social benefit indexation schemes are insufficient to automatically offset potential (pre-revenue-recycling) welfare losses of vulnerable households. This is not surprising, as consumer price changes are concentrated in energy commodities and exceed changes of the broader overall consumption price or wage indices. Our results therefore indicate a need for policymakers to rethink the functioning of the social welfare system and to take into account its interaction with the tax system in the light of ambitious climate policy when lump-sum recycling of revenues is not feasible. Ensuring a fair transition to climate neutrality while reducing energy poverty may therefore require (a broadening and deepening of) targeted measures to complement existing systems for social protection. We note here that several measures directed at low-income households are already in place in the EU Member States, including ‘social tariffs’ (lowered energy prices for particular population subgroups) and means-tested subsidies for investments in residential energy efficiency, such as the ‘Habiter Mieux’ initiative in France and the ‘Stromspar-Check’ project in Germany. These detailed measures are not included in the analysis here, but more research on their efficiency and distributional impacts is welcome, particularly in the context of the Social Climate Fund and the Social Climate Plans as proposed by the European Commission in the Fit for 55 package to deliver the European Green Deal (EC, 2021).

Second, transfer indexation exacerbates (pre-revenue-recycling) welfare losses for households relying on social benefits when this indexation is tied to wages that decline relative to the baseline (Fig. 5a and c). For wage indexing, the sign of welfare loss offsetting differs across countries due to the macro results (Table 2): wages in most countries drop relative to baseline levels due to climate policy, while wages in France and Belgium see an upward evolution (also in Austria and Spain under Partial Auctioning). We find that CPI-based indexation schemes generally offer somewhat better protection to impacts stemming from carbon pricing than wage-based indexation. However, some countries, such as Belgium, explicitly exclude fuel prices from the index used to update social transfers, which could limit the automatic climate policy impact offsetting mechanism in real-world applications.

**Fig. 6:**
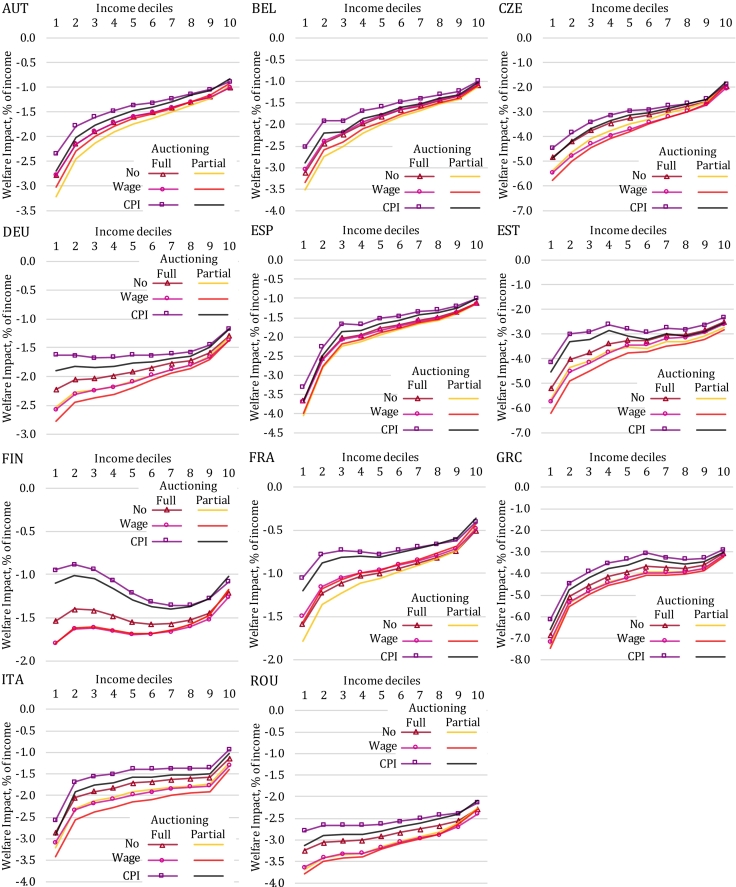
Sensitivity of pre-revenue-recycling welfare impacts to permit auctioning and social benefit indexation options. Source: EUROMOD-ITT extension.

Third, although the regressive (pre-revenue-recycling) impact pattern is somewhat mitigated under CPI-based indexation of social transfers, it remains robust to the stylized variations in transfer indexation schemes we study here, unlike results from recent studies for the US (Goulder et al., 2019; Cronin et al., 2019). For most countries, the gains from indexation are of comparably small magnitude, and are not necessarily highly concentrated in the bottom deciles of the income distribution. Pensions represent an important social benefit category in many countries, hence the shape of the curves displayed in Fig. 5 will be influenced by the position of the pensioners in the income distribution. The relative importance of transfer types, e.g. pensions relative to unemployment benefits, is another factor affecting the impact pattern of indexing across income groups. For the majority of countries, we find a regressive pre-revenue-recycling welfare impact pattern across all six scenarios considered. The comparison across scenarios by Member State presented in Fig. 6 illustrates the sensitivity of the impact pattern to climate policy design (full vs. partial auctioning) and social benefit indexation schemes. Estonia, for instance, is one example with a relatively large increase in the CPI (see Table 2) and with gains from social transfer indexation skewed towards low-income households. As a result, the distribution of welfare impacts becomes roughly uniform (apart from bottom and top deciles) when we account for CPI-based indexation, whereas the no-indexation benchmark indicated a regressive pattern over the entire income distribution. The fall in wages compared to the baseline is also relatively strong in Estonia, such that a wedge emerges between the impact patterns for the various indexation cases. Findings for Germany are similar, and for Finland the impact pattern even turns progressive. These results highlight the importance of changes in transfer income when estimating the distributional impacts of climate policies, and are therefore informative for future modelling efforts.

## 5 Conclusion

In order to ensure broad societal support for ambitious climate policy, reforms need to strike a balance between several objectives. Ultimately, it is up to policymakers to decide how to design policy measures that are acceptable to stakeholders. However, models can assist policymakers in revealing the potential impact of certain choices. Here, we attempt to quantify the impact of various climate policy design choices on competitiveness and equity, two important topics that are typically discussed separately.

Results illustrate that expenditure-side impacts of carbon pricing tend to be regressive, echoing earlier work. The magnitude, pattern and drivers of welfare impacts differ across countries in the EU. Our decomposition shows that, for most countries, residential energy use is the main driver for this result. Importantly, an across-the-board lump sum reallocation of carbon dividends offsets the regressive pattern and turns the tax reform into a progressive one. Our scenarios assume that this transfer is handed out equally to all households, also those in the higher income groups. Cleary, this is a stylised assumption, and targeted revenue recycling to low-income households and those facing energy poverty could be a more effective way to ensure a just transition. This could, at the same time, leave room for revenue recycling to support other elements of a policy package for a transition towards carbon neutrality, such as reducing labour taxation or supporting green investment and R&D.

Furthermore, our results show that other elements of climate policy design may affect the distributional consequences. Grandfathering permits to energy-intensive trade-exposed industries reduces their output losses but tends to strengthen the regressivity of climate policy for three reasons. First, carbon taxes are higher as higher output levels need to be reconciled with a fixed cap on emissions, putting larger emphasis on the expenditure-side effects. Higher prices enhance regressivity particularly when a uniform carbon price applies to both industry and households, or when both are covered under one and the same emission trading scheme. As such, this channel may become increasingly relevant when countries move towards integrated and comprehensive carbon pricing schemes. Second, tax revenue tends to be reduced when part of the permits is handed out for free, which limits the extent to which lump sum transfers can offset the welfare losses for low-income households. Interestingly, and perhaps counterintuitive, our analysis also shows instances where tax revenue goes up despite a narrower tax base, highlighting the importance of sector composition and fiscal federalism aspects in EU climate policy. Third, grandfathering permits raises capital incomes, which are typically concentrated in higher income groups. Regardless, due to the size of these effects, the distributional patterns for Full and Partial Auctioning are very similar in our scenarios. While our results reveal trade-offs between equity and competitiveness, they suggest that ample policy space exists to reconcile both dimensions. Alternative ways to safeguard the competitive position of EU industrial sectors, such as carbon border adjustment mechanisms, may overcome some of the trade-offs as they generate more, not less revenue.

Finally, our scenarios show that the characteristics of the welfare system in a country can influence the distributional effects of carbon pricing. Indexing existing social benefits to consumption prices that rise under climate policy tends to mitigate the welfare losses for the bottom half of the income distribution, although the magnitude of this effect is insufficient to counterbalance regressive expenditure-side effects. This indicates that while interactions with the broader welfare system should be considered, automatic benefit indexation schemes alone cannot ensure an equitable transition to a low-carbon society.

This paper sets up a methodological framework that combines economy-wide sector-specific outputs with detailed household-level data on income and expenditures. Although the framework can be improved in several ways, this kind of modelling toolbox can be a valuable asset in analysing policy-relevant scenarios in the areas of climate policy and energy taxation. Further work could highlight the sensitivity of results with respect to particular assumptions made in reconciling micro and macro data. In addition, scenarios could implement a mixed policy design that combines targeted transfers to low-income households with measures aimed at improving competitiveness, such as reducing labour taxes or grandfathering permits. Finally, an important caveat of the work presented here is the limited detail on the income side. We assume that higher wages equally benefit all workers, and workers only. This implies that we disregard the extensive labour market margin, whereas additional labour demand could well benefit lower income groups by shifting from unemployed to employed. Our results also indicate that climate policy impacts are likely concentrated in particular sectors, which would give rise to high income impacts for a narrow group of workers. Illustrating these effects is an important avenue for future work, as well as assessing alternative measures for carbon leakage prevention, such as border carbon adjustments.

## Author contributions

MW performed CGE analysis. SR and AM performed microsimulation analysis. SR, AM and TV performed benefit indexation sensitivity analysis. LR and KW prepared CGE baseline and model calibration. TV designed the research and wrote the paper. All authors contributed to the analysis and writing of the paper.

## Appendix A Supplementary data

Supplementary material Image 1
