# Supplementary material for: Climate policy design, competitiveness and income distribution: A macro-micro assessment for 11 EU countries
Source: Energy Econ. 2021 Nov;103:None. doi: 10.1016/j.eneco.2021.105538 (PMC8594799; doi:10.1016/j.eneco.2021.105538)
Supplement: Supplementary file 1 — Supplementary material [file mmc1.pdf]

Supplementary Information

To assess whether these 11 countries make up a representative sample of all EU Member States, Supplementary Figure 1 plots 2018 values of two key variables relevant for the current analysis: the share of residential energy in total household consumption expenditures and the share of non-renewable energy in heating and cooling energy. The 11 countries that are covered do not show a particular bias along these variables. However, since a few outlier Member States are not included in our study, the results may underestimate the full impact variation across countries in the EU.

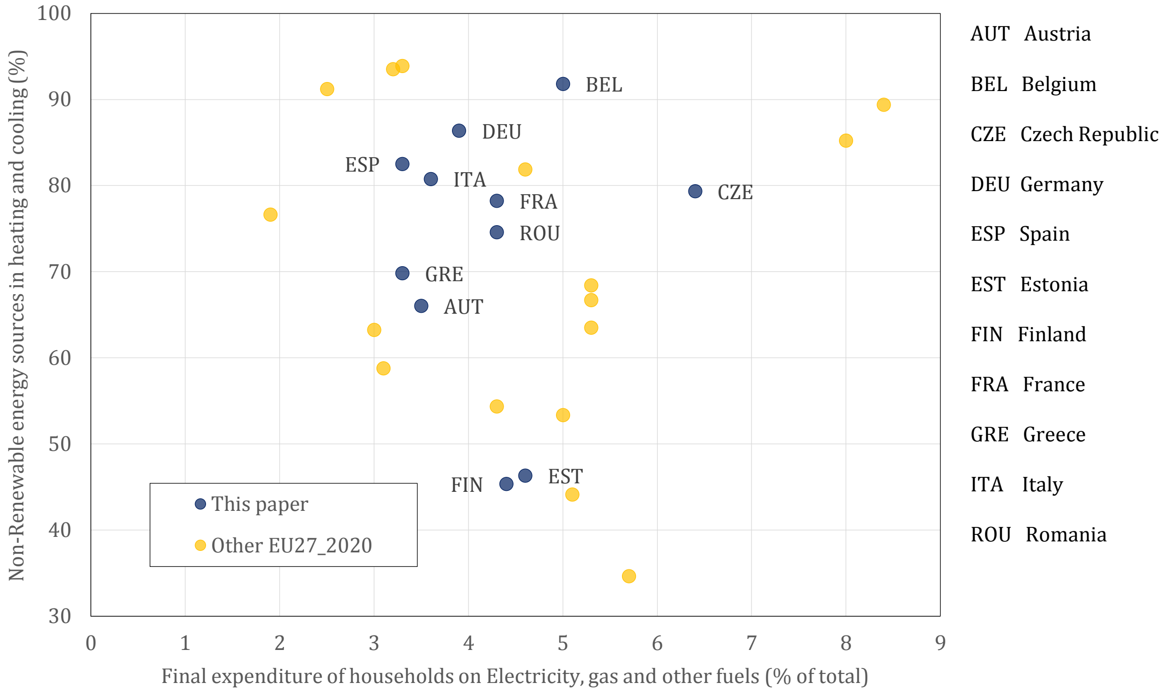

Supplementary Figure 1: Country coverage and corresponding energy use characteristics (Eurostat, Data for 2018; Data for 2017 was used for Greece).

Supplementary Table 1: Mapping between consumption categories in both models

JRC-GEM-E3		EUROMOD-ITT	
1	Food, beverages and tobacco	1	Food and non-alcoholic beverages
		2	Alcoholic beverages
		3	Tobacco
2	Clothing and footwear	4	Clothing and footwear
3	Housing and water charges	6	Rents (excluding imputed rents)
4	Fuels and power	5	Home fuels, electricity and water
6	Heating and cooking appliances	7	Household goods and services
7	Medical care and health	8	Health
5	Household equipment and operations	98	Durable goods
8	Purchase of vehicles		
9	Operation of personal transport equipment	9	Private transport
10	Transport services	10	Public Transport
11	Communication	11	Communication
12	Recreational services	12	Recreation and culture
		14	Restaurants and hotels
13	Miscellaneous goods and services	15	Other goods and services
14	Education	13	Education
